# Supplementary material for: Exploring the Molecular Basis of Vesicular Stomatitis Virus Pathogenesis in Swine: Insights from Expression Profiling of Primary Macrophages Infected with M51R Mutant Virus
Source: Pathogens. 2023 Jun 30;12(7):896. doi: 10.3390/pathogens12070896 (PMC10384765; doi:10.3390/pathogens12070896)
Supplement: Supplementary file 1 [file pathogens-12-00896-s001.zip › pathogens-2407929-supplementary.pdf]

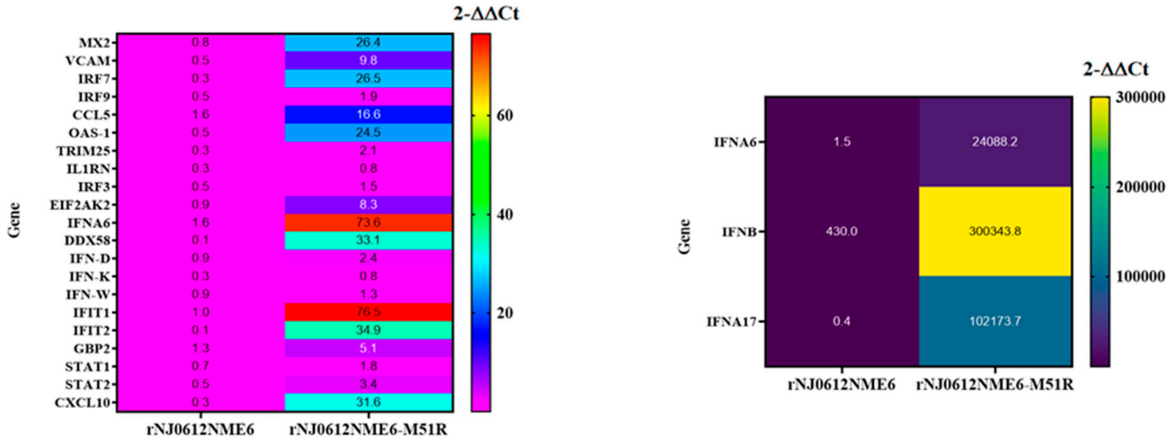

Figure S1. qPCR validation of some relevant genes.

Group	Gene	EXP	M/W	FDR	M/N	FDR
IFNs	IFNAs * (18)	92	22.8	0.00	23.7	0.00
	IFNB	179	6.9	0.01	42.5	0.00
	IFND	94	8.5	0.00	11.5	0.00
	IFNK	69	1.5	0.03	1.5	0.10
	IFNW	62	2.2	0.00	1.9	0.03
	IFNG	205	-1.4	0.89	1.7	0.88
	IFNL	147	10.7	0.00	13.0	0.00
IFN signaling pathway	IFNAR1	3774	2.3	0.01	-1.3	0.77
	IFNAR2	2223	8.0	0.00	2.8	0.08
	IFNGR1	1000	2.0	0.04	-1.3	0.75
	JAK2	215	4.4	0.00	3.2	0.00
	IRF9	634	5.5	0.00	3.2	0.00
	STAT1	4650	1.6	0.02	1.8	0.04
	STAT2	2794	4.5	0.00	5.2	0.00

Table S1. Expression levels (EXP), false discovery rates (FDR) and fold differences (mutant vs WT, M/W and mutant vs mock infection, (M/N) of interferon related genes differentially expressed by infected macrophages. * The results were the average of 17 IFNA probes.

Group	Gene	EXP	M/W	FDR	M/N	FDR
Viral RNA senser or enhancer	MDA5	231	44.1	0.00	74.2	0.00
	RIG-I	154	24.5	0.00	21.6	0.00
	ZNFX1	1500	14.2	0.00	10.1	0.00
	TRIM25	296	6.3	0.00	5.2	0.00
Signal transducer	MAVS/IPS1	452	1.8	0.03	-1.1	0.88
	TBK1	459	3.0	0.00	1.5	0.46
	IKBKE	1575	1.9	0.02	1.3	0.72
Transcription factor	IRF3	194	1.7	0.13	1.6	0.37
	IRF5	3796	2.2	0.00	1.0	0.98
	IRF7	435	5.2	0.00	5.6	0.00
	IRF8	2039	4.5	0.00	3.3	0.01
IFN expression inhibitory gene	AHR	193	-6.0	0.00	1.0	1.00
	DUSP1	2208	-13.9	0.00	-1.0	1.00
	HES1	218	-14.0	0.00	-1.3	0.92
	PRDM1	158	-4.1	0.02	1.5	0.81
NFκB inhibitory gene	FOS	1993	-18.0	0.00	1.3	0.94
	FOSL2	790	-5.0	0.00	1.2	0.91

Table S2. Expression levels (EXP), false discovery rates (FDR) and fold differences (mutant vs wildtype, M/W and mutant vs mock infection, M/N) of differentially expressed genes in cytosolic pattern recognition receptor signaling pathways or with inhibitory effect on IFN expression.

Group	Gene	EXP	M/W	FDR	M/N	FDR
IFN signaling suppressor	SOCS1	549	11.6	0.00	7.2	0.02
	SOCS3	9021	2.1	0.01	2.3	0.02
	USP18	1176	12.4	0.00	9.3	0.01
RIG-I-like signaling suppressor	IRF2	323	5.7	0.00	3.6	0.01
	NLRC5	368	5.0	0.00	4.3	0.00
	TRIM13	322	3.0	0.04	-1.2	0.89
	TRIM21	149	4.9	0.00	4.3	0.01
	TRIM26	1024	7.5	0.00	3.4	0.01
	TRIM38	1448	5.7	0.00	2.8	0.01

Table S3. Expression levels (EXP), false discovery rates (FDR) and fold differences (mutant vs WT, M/W and mutant vs mock infection, M/N) of differentially expressed genes with suppressive effects on IFN and RIG-I-like signaling.

Group	Gene	EXP	M/W	FDR	M/N	FDR
Cytokines	CSF2	273	-5.8	0.04	-1.0	0.99
	IL1RN	922	22.7	0.00	23.8	0.02
	IL15	162	6.2	0.00	12.1	0.00
	IL18	651	5.1	0.01	8.1	0.01
	IL27	318	3.2	0.01	12.1	0.00
	LIF	217	-5.2	0.02	2.5	0.50
	TNFSF13B	1506	8.2	0.00	4.8	0.00
	IL1B	2568	3.5	0.39	25.3	0.05
	IL10	1109	-1.0	0.98	11.1	0.00
Cytokine receptors	IL1R2	219	-2.5	0.04	1.2	0.92
	IL1RAP	508	3.8	0.01	1.5	0.69
	IL4R	3136	2.4	0.04	1.0	1.00
	IL7R	471	9.6	0.00	21.7	0.00
	IL15RA	377	7.8	0.00	5.7	0.03
	IL20RB	92	-2.2	0.04	1.2	0.85
	IL10RA	672	5.1	0.00	3.0	0.01

Table S4. Expression levels (EXP), false discovery rates (FDR) and fold differences (mutant vs WT, M/W and mutant vs mock infection, M/N) of cytokine, chemokine and the receptor genes differentially expressed by infected macrophages.

Group	Gene	EXP	M/W	FDR	M/N	FDR
CCLs	CCL3	2336	-1.1	0.94	6.7	0.03
	CCL4	3028	1.4	0.79	42.1	0.00
	CCL5	6487	1.6	0.50	4.8	0.02
	CCL20	412	1.6	0.75	28.5	0.01
	CCL8	5867	5.5	0.03	6.2	0.09
ELR+ CXCLs	CXCL1	1791	-7.2	0.01	-1.1	0.97
	CXCL2	3008	-6.1	0.02	3.5	0.34
	CXCL3	2388	-4.6	0.03	1.5	0.84
	CXCL5	1786	-1.4	0.03	-1.1	0.89
	CXCL6	1723	-1.4	0.07	1.0	0.96
	CXCL8	2000	2.4	0.36	13.2	0.02
	ELR+CXCLs *	12695	-2.3	n/a	1.9	n/a
ELR- CXCLs	CXCL9	194	12.2	0.00	15.7	0.00
	CXCL10	10463	54.3	0.00	15.3	0.16
	CXCL11	126	52.5	0.00	43.0	0.00
	CXCL(9,10,11) *	10783	50.6	n/a	15.4	n/a
	CXCL13	70	2.1	0.00	2.3	0.01
	CXCL14	2061	1.6	0.05	-1.0	0.96
	CXCL16	5676	1.7	0.02	1.9	0.04
Chemokine receptors	CCR5	246	6.5	0.00	2.3	0.10
	CCR7	206	-4.3	0.00	1.0	0.98
	CCRL2	1839	7.0	0.00	4.0	0.00
	CXCR4	1060	-10.8	0.00	-2.5	0.23

Table S5. Expression levels (EXP), false discovery rates (FDR) and fold differences (mutant vs WT, M/W and mutant vs mock infection, M/N) of chemokine and receptor genes differentially expressed by infected macrophages. *[1] The results were the sums of the gene groups, and the fold differences were normalized with gene expression levels.

MHC	Group	Gene	EXP	M/W	FDR	M/N	FDR
Class I antigen processing	Ubiquitin	AREL1	276	2.8	0.01	1.1	0.96
		RBBP6	101	2.4	0.00	2.3	0.00
		UBA7	265	2.0	0.05	4.1	0.00
		UBE2A	1334	1.5	0.00	1.5	0.04
		UBE2D1	165	5.7	0.00	5.1	0.00
		UBE2H	696	3.2	0.00	2.5	0.02
		UBE2B	841	1.6	0.01	1.4	0.36
		UBE2K	142	1.6	0.00	-1.1	0.73
		UBE3A	192	2.2	0.04	-1.1	0.95
	Proteasome	PSMA5	2805	2.6	0.00	1.6	0.41
		PSMA6	6247	2.1	0.01	1.9	0.08
		PSMB2	3866	1.6	0.01	1.4	0.21
		PSMB5	159	-1.7	0.05	-1.1	0.94
		PSMD6	1372	1.9	0.01	1.1	0.86
		PSMD9	373	1.5	0.03	-1.0	0.99
		PSME4	161	2.3	0.01	1.0	0.97
	Transporter	TAP1	1295	2.8	0.01	4.2	0.00
		TAP2	719	1.4	0.07	2.3	0.00
		TAPBP	1514	-1.3	0.28	1.9	0.01
Class II antigen processing	Cathepsin	CTSB	284	-5.7	0.00	-1.3	0.88
		CTSC	1863	4.0	0.00	1.2	0.87
		CTSH	56769	1.4	0.01	-1.0	0.97
Antigen presentation	Th activation	TNFSF18	87	2.3	0.00	2.2	0.03
	Coactivator	CD40	1525	4.2	0.00	4.1	0.01

Table S6. Expression levels (EXP), false discovery rates (FDR) and fold differences (mutant vs WT, M/W and mutant vs mock infection, M/N) of chemokine and receptor genes differentially expressed by infected macrophages.

Group	Gene	EXP	M/W	FDR	M/N	FDR
Apoptosis	BAK1	395	2.9	0.02	1.5	0.67
	BCL2L13	1167	2.0	0.00	-1.0	0.96
	BIK	82	2.6	0.00	2.3	0.01
	CASP3	685	4.4	0.00	2.2	0.10
	CASP4	1204	1.9	0.03	2.2	0.03
	CASP7	651	2.9	0.00	3.7	0.00
	CASP8	337	4.4	0.00	2.1	0.03
	CASP10	98	6.0	0.00	6.1	0.00
	CASP15	92	3.3	0.00	3.8	0.00
	DAPK1	246	2.0	0.00	-1.1	0.94
	DAXX	2794	5.9	0.00	3.5	0.03
	DIDO1	162	2.0	0.01	-1.1	0.95
	XAF1	314	13.1	0.00	11.1	0.00
Death receptor signaling	TNF	2641	1.1	0.97	25.5	0.00
	TNFSF10/TRAIL	179	80.5	0.00	46.7	0.00
	TNFSF15/TL1A	214	5.6	0.00	3.5	0.06
	TNFRSF1A/TNFR1	3943	2.3	0.00	1.0	0.98
	TNFRSF1B/TNFR2	2987	-1.8	0.01	-1.9	0.02
	TNFRSF3/LTBR	8211	2.5	0.01	-1.4	0.61
	TNFRSF6/FAS	181	3.3	0.00	3.5	0.01
	TNFRSF10/DR5	203	3.1	0.00	8.4	0.00
	FADD	145	3.0	0.00	1.3	0.68
	RIPK1	1864	2.9	0.00	1.4	0.48
	TRAF2	305	2.6	0.03	2.9	0.09
Anti-apoptosis	BIRC3	656	2.6	0.01	9.1	0.00
	REL	100	-3.2	0.03	1.5	0.77

Table S7. Expression levels (EXP), false discovery rates (FDR) and fold differences (mutant vs WT, M/W and mutant vs mock infection, M/N) of apoptosis related genes differentially expressed by infected macrophages.
